# Prospective Evaluation of Three Rapid Diagnostic Tests for Diagnosis of Human Leptospirosis

**DOI:** 10.1371/journal.pntd.0002290

**Published:** 2013-07-11

**Authors:** Marga G. A. Goris, Mariska M. G. Leeflang, Martin Loden, Jiri F. P. Wagenaar, Paul R. Klatser, Rudy A. Hartskeerl, Kimberly R. Boer

**Affiliations:** 1 KIT Biomedical Research, Royal Tropical Institute (KIT), Amsterdam, The Netherlands; 2 Department of Clinical Epidemiology and Biostatistics, Academic Medical Center, Amsterdam, The Netherlands; 3 Department of Global Health, Academic Medical Center, Amsterdam, The Netherlands; Universidad Peruana Cayetano Heredia, Peru

## Abstract

**Background:**

Diagnosis of leptospirosis by the microscopic agglutination test (MAT) or by culture is confined to specialized laboratories. Although ELISA techniques are more common, they still require laboratory facilities. Rapid Diagnostic Tests (RDTs) can be used for easy point-of-care diagnosis. This study aims to evaluate the diagnostic performance of the RDTs LeptoTek Dri Dot, LeptoTek Lateral Flow, and Leptocheck-WB, prospectively.

**Methodology:**

During 2001 to 2012, one or two of the RDTs at the same time have been applied prior to routine diagnostics (MAT, ELISA and culture) on serum specimens from participants sent in for leptospirosis diagnosis. The case definition was based on MAT, ELISA and culture results. Participants not fulfilling the case definition were considered not to have leptospirosis. The diagnostic accuracy was determined based on the 1^st^ submitted sample and paired samples, either in an overall analysis or stratified according to days post onset of illness.

**Results:**

The overall sensitivity and specificity for the LeptoTek Dri Dot was 75% respectively 96%, for the LeptoTek Lateral Flow 78% respectively 95%, and for the Leptocheck-WB 78% respectively 98%. Based on the 1^st^ submitted sample the sensitivity was low (51% for LeptoTek Dri Dot, 69% for LeptoTek Lateral Flow, and 55% for Leptocheck-WB), but substantially increased when the results of paired samples were combined, although accompanied by a lower specificity (82% respectively 91% for LeptoTek Dri Dot, 86% respectively 84% for LeptoTek Lateral Flow, and 80% respectively 93% for Leptocheck-WB).

**Conclusions:**

All three tests present antibody tests contributing to the diagnosis of leptospirosis, thus supporting clinical suspicion and contributing to awareness. Since the overall sensitivity of the tested RDTs did not exceed 80%, one should be cautious to rely only on an RDT result, and confirmation by reference tests is strongly recommended.

## Author Summary

Leptospirosis is one of the world's most spread zoonoses causing acute fever. The illness can rapidly develop into a severe, potentially fatal, form with a high mortality rate. Laboratory tests are needed to confirm the diagnosis. Culturing leptospires from patient material can take months to grow. Therefore, most used laboratory tests are based on detection of antibodies against leptospires. The microscopic agglutination test is considered the reference standard but is only performed at specialized laboratories. In this study, we measured the diagnostic accuracy of three rapid diagnostic tests (RDTs) by doing a prospective evaluation during 11 years. These tests produce results within 15 minutes. The overall sensitivities (77%) and specificities (96%) were similar for the RDTs. Evaluating the first submitted specimen resulted in lower sensitivities (51% for LeptoTek Dri Dot, 69% for LeptoTek Lateral Flow, and 55% for Leptocheck-WB). When paired specimens were evaluated, the sensitivity increased although the specificity decreased (82% respectively 91% for LeptoTek Dri Dot, 86% respectively 84% for LeptoTek Lateral Flow, and 80% respectively 93% for Leptocheck-WB). Based on these results confirmation by reference tests is still strongly recommended, although the RDTs contribute to the diagnosis of leptospirosis, thus supporting clinical suspicion and contributing to awareness.

## Introduction

Leptospirosis is caused by microorganisms of the genus *Leptospira*. It is one of the world's most wide-spread zoonoses, with a mean global incidence of endemic and epidemic leptospirosis of 5 per 100,000 and 14 per 100,000 population, respectively [1]. It causes an acute febrile illness [2] with a wide diversity of milder clinical signs such as headache, malaise, myalgia, conjunctival suffusion and sometimes a transient rash. However, the illness can rapidly develop into a severe, potentially fatal form with a high mortality rate [3]. Leptospirosis is often overlooked since it mimics many other diseases, including dengue, malaria, influenza and hantavirus infections [4], making differential diagnosis very difficult based on clinical grounds alone. Laboratory tests are therefore the basis of a confirmed case of leptospirosis.

The most commonly used laboratory tests are based on detection of antibodies against the leptospires. Pathogenic leptospires enter the body through small cuts or abrasions, or via mucous membranes and possibly through wet skin. After infection, leptospires circulate in the blood stream, with a bacteremic phase lasting for up to 10 days post onset of the disease (DPO). Detectable antibodies appear in the blood about 5–10 DPO [5], and sometimes later, especially if antibiotic treatment is instituted [4]. These antibodies can be detected by a variety of laboratory assays such as the microscopic agglutination test (MAT), enzyme-linked immunosorbent assay (ELISA) and indirect fluorescent antibody test (IFAT) [6]. Currently, the MAT is considered the reference standard in serodiagnosis and as such has a worldwide application. However, MAT and ELISA are technically demanding and relatively expensive tests and therefore not widely applicable in peripheral healthcare facilities, especially in tropical and subtropical developing regions where leptospirosis is most endemic. Culturing leptospires out of blood provides proof of infection but is insensitive [7] and has little clinical value for patient management as it can take weeks to months to confirm results. PCR on blood has proven to be useful in the first week of the disease [8], however many laboratories are not equipped to run PCR tests. Hence, for most clinical situations rapid diagnostic tests (RDTs) can play an important role in immediate case detection and clinical management. Most commonly used RDTs are based on the immunochromatographic lateral flow technology.

To date, a variety of RDTs have been described and evaluated in various papers [9]–[11], most of these being short term retrospective evaluations and often concern evaluations of a single RDT. Our aim was to evaluate the diagnostic accuracy of three RDTs, applied on serum specimens from suspected leptospirosis patients from The Netherlands in a prospective cohort of leptospirosis suspected patients. Additional aims were to assess whether there are differences between the three tests and whether using the tests at different times since patient's onset of symptoms leads to differences in diagnostic accuracy. The RDTs used in this research were available in the Netherlands, or could be easily imported.

## Materials and Methods

Standards for the Reporting of Diagnostic accuracy testing (STARD) checklist were adhered to throughout the text (Table S1) [12].

### Study participants

**Figure 1:**
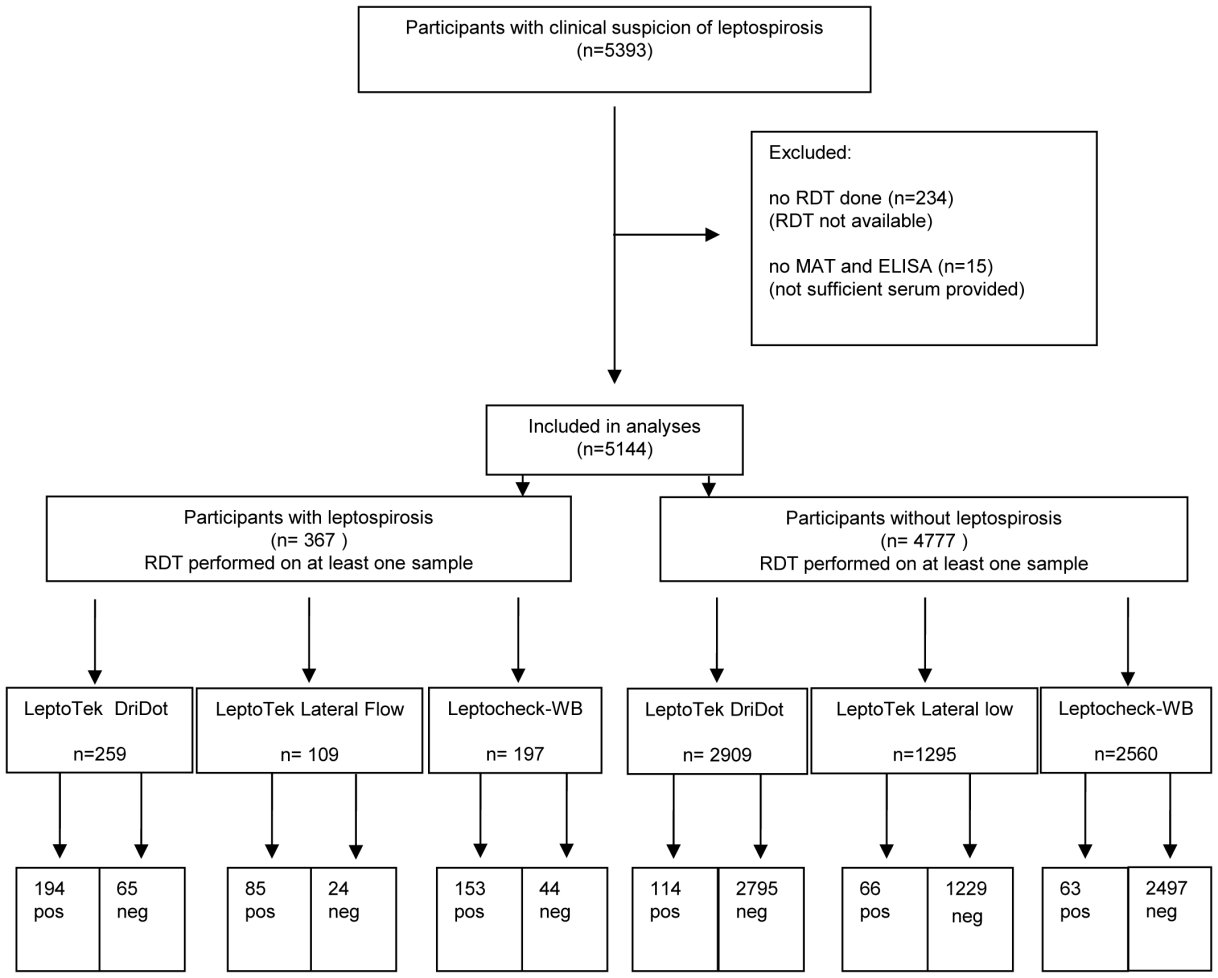
Flow chart of participants and rapid diagnostic tests.

The Royal Tropical Institute (KIT), Biomedical Research houses the WHO/FAO/OIE and National Collaborating Centre for Reference and Research on Leptospirosis (NRL), which confirms about 99% of the suspected cases of leptospirosis in The Netherlands. The typical annual number of suspected cases is around 500, of which approximately 30 are confirmed leptospirosis cases. About 50% of the confirmed cases have contracted leptospirosis during travel abroad. In the period of evaluation, July 2001 to August 2012, the population in The Netherlands was stable at about 16 million. During this period, all human blood specimens sent by physicians practicing in the Netherlands to NRL for leptospirosis diagnosis were tested upon arrival by routine diagnostics. In most cases only one sample was received per participant, in other cases two or more samples. Further inclusion and exclusion criteria of samples and participants are depicted in a flow diagram (Figure 1). Laboratory tests routinely performed are MAT and in-house IgM-ELISA. Culture was done as described below. A single or a combination of two RDTs were prospectively performed for evaluation purposes.

### Leptospirosis case definition

Patients were considered as having leptospirosis based on one or more of the following criteria: (i) single MAT titer with a pathogenic strain ≥1∶160, (ii) single IgM-ELISA titer ≥1∶160, (iii) positive culture or (iv) seroconversion/≥four-fold titer rise MAT or IgM ELISA (titer ≤1∶20 to ≥1∶80) in paired samples taken at least 2 days apart [13]. The treating physician was encouraged to send multiple samples for laboratory testing for all participants.

### Laboratory methods

RDTs were applied prior to and independent of routine diagnostic testing. All tests were performed by skilled staff of NRL (10 persons) who followed detailed protocols about interpretation of tests. NRL is accredited based on ISO 15189 since 2006. All serological tests were performed on serum specimens which were inactivated in a 56°C water bath for 30 minutes before testing.

#### Culture

Culture was initiated for blood, plasma or serum samples collected within the first 10 days of disease. Urine was cultured at all time points during the course of disease within 2 hours after voiding. Fletcher medium and Ellinghausen-McCullough as modified by Johnson and Harris (EMJH) culture medium was used [14]. EMJH was supplemented with 5-fluorouracil (200 µg/ml), 1% (V/V) rabbit serum and 1% (V/V) fetal calf serum or combinations [15]. Inoculated media were incubated for a maximum of 4 months at 30°C and (bi)weekly checked for leptospiral growth by darkfield microscopy.

#### Microscopic agglutination test

The MAT was performed with a panel of live leptospires as described elsewhere [15]. The panel consisted of 16 strains of the pathogenic serovars Bratislava, Ballum, Canicola, Grippotyphosa, Hebdomadis, Icterohaemorrhagiae, Copenhageni, Poi, Pomona, Proechimys, Hardjo, Saxkoebing and Sejroe, and the non-pathogenic serovar Patoc. Sera from patients who visited a country outside the Netherlands within one month prior to the day of onset of symptoms were also tested with an additional panel of 12 globally representative strains, i.e. the pathogenic serovars Australis, Rachmati, Bataviae, Celledoni, Cynopteri, Mini, Panama, Pyrogenes, Shermani and Tarassovi, and the non-pathogenic serovars Andamana and Semaranga.

#### IgM ELISA

In-house developed ELISA for the detection of *Leptospira*-specific IgM antibodies (IgM ELISA) was performed with antigen prepared from the local strain Wijnberg (serovar Copenhageni, serogroup Icterohaemorrhagiae) [16], [17].

#### Rapid diagnostic tests

Three rapid diagnostic serological tests were used according to their availability: (i) 2001–2008 LeptoTek Dri Dot, Organon Teknika B.V., later bioMérieux B.V., Boxtel, the Netherlands. (ii) 2001–2004 LeptoTek Lateral Flow, Organon Teknika B.V. Boxtel, the Netherlands. (iii) 2004–2012 Leptocheck-WB, Zephyr Biomedicals, Verna Goa, India. LeptoTek Lateral Flow and Leptocheck-WB are lateral flow immunochromatographic tests. These are both qualitative, sandwich immunoassays intended for the detection of *Leptospira*-specific IgM antibodies in humans. The test can be read after 10 to 15 minutes and can be used for serum/plasma or whole blood specimens. LeptoTek Dri Dot is a latex agglutination assay and detects *Leptospira*-specific antibodies (IgM and IgG) in human sera.

The rapid tests were performed according to the manufacturer's instructions. For the LeptoTek Lateral Flow 5 µl serum was spotted in the sample port of the device, running buffer was added and the test was read after 10 minutes. For the Leptocheck-WB 10 µl serum was spotted in the sample port of the device and 15 minutes after running buffer was added the test was read. Both tests were valid when the control band stained. Valid tests were scored positive when a test band was observed, negative when no band was observed and indeterminate when it was unclear whether a band was observed or not. Invalid tests were repeated. For the LeptoTek Dri Dot 10 µl of serum was mixed with the dried leptospiral-antigen-coated latex spot on the agglutination card. The test was read within 30 seconds and scored positive when agglutination was observed, negative when there was no agglutination and indeterminate when occurrence of agglutination was unclear.

### Data analyses

Data were entered into a Laboratory Information System (LASSIST, Mechatronics Software Applications BV, the Netherlands) and exported and analyzed in SPSS (version 19, IBM, NY, USA). These included patient data obtained from the request form (i.e. gender, date of birth, date of onset, travel history). The results of each diagnostic test of every sample were entered into the database. Follow-up samples taken less than two days after the first sample were excluded. Indeterminate results were regarded as negative, unless otherwise stated.

#### Overall accuracy

In this analysis, the overall accuracy of RDTs for diagnosing leptospirosis for any submitted sample was estimated. Diagnostic accuracy was defined by sensitivity and specificity with 95% confidence intervals (CIs) [18]. For these analyses, participants were considered positive if they had a positive RDT result in at least one of the submitted samples (participant-level, not on individual samples received). Sensitivity was calculated in participants who fulfilled the case-definition, specificity on those who did not. The three RDTs were considered different from each other if the 95% confidence intervals did not overlap.

#### Overall accuracy – First sample sent in and follow-up sample

To avoid potential overestimations of sensitivity and underestimations of specificity of the individual test in the above analyses, a subgroup analysis was completed on only the first sample that was sent in, and, if available, on the follow-up sample (paired samples), if taken within 1 month. This reflects clinical practice better than the previous analysis, as it represents the disease period when leptospirosis diagnostics are typically requested by the clinician. As well, this analysis does not depend on a defined first day of illness. The three tests were considered different from each other if the 95% confidence intervals did not overlap.

#### Time trends

For those patients with data available on their first day of illness (50%), the diagnostic accuracy of the serologic tests was calculated at different time-periods, i.e., 0–4 days post onset of symptoms (DPO) (early acute), 5–10 DPO (late acute), 11–20 DPO (convalescent) and >20 DPO (late convalescent). If multiple samples of a participant were taken in the same time-period, the sample with the lowest DPO was included.

#### Sensitivity analyses

A substantial proportion of the samples were scored indeterminate in the RDTs. To assess the impact of the interpretation of indeterminate results as considered negative in the previous analyses, a sensitivity analysis of the diagnostic accuracy was conducted by allocating the indeterminate scores to either the negative test results or positive test results or by excluding these indeterminate scores for 1^st^ and follow-up samples.

Furthermore the predictive value of an indeterminate versus a negative test result was assessed: from participants whose first test result was either indeterminate or negative, we looked at the RDT result in the follow-up sample to calculate the proportion of participants fulfilling the case definition. This denotes the proportion of patients changing from a negative or indeterminate RDT to a positive RDT.

An additional analysis was completed to determine the potential differences in diagnostic accuracy of the RDTs between infecting serogroups. This analysis considered infections with serogroup Icterohaemorrhagiae, Grippotyphosa, other serogroups and not classifiable serogroups for the 1^st^ sample and paired samples when available.

To investigate the consistency of the diagnostic accuracy of these RDTs through the periods of use, sensitivity and specificity were compared for each diagnostic test for the 1^st^ sample and paired samples by years the test was completed.

### Ethical statement

This data collection was exempted from ethical review of human subjects research by the Medical Ethical Review Committee of the Academic Medical Centre, University of Amsterdam (W12_076#12.17.0092). All data presented have been de-identified and were not attributable to individual patients.

## Results

During the 11 years of data collection, blood specimens from 5393 participants suspected of leptospirosis were submitted to NRL for testing. The majority of participants (95.4%) were tested by MAT, IgM ELISA and one or more of the rapid tests (Figure 1); however there were short periods where no RDT could be performed due to their unavailability on the international market (Table S2). No RDT could be completed for 234 participants. Furthermore, 15 participants were excluded as there was no MAT or ELISA completed, as a prerequisite of the reference standard and case definition, leaving a total of 5144 patients. Follow-up specimens were received from 929/5144 participants and 53.1% of the participants had a documented DPO.

There were 367 (6.7%) leptospirosis cases fulfilling the case definition, with a male to female sex ratio of about 6∶1. The sex ratio of non-leptospirosis cases was 2∶1. The mean age of cases and non-cases was 39.7 and 42.1 years, respectively. Male leptospirosis cases were older (mean age 40.2, SD 15.7) than female cases (mean age 36.8, SD 17.3). Table 1 presents an overview of characteristics of the eligible study participants. Table 2 presents an overview of the participants fulfilling the case definition. There were no invalid test results reported for the RDTs, i.e. the control band in the LeptoTek Lateral Flow and Leptocheck-WB stained in all tests performed.

**Table 1:** Characteristics of participants.

Characteristic	Total	Leptospirosis patients	Non leptospirosis patients
	(n = 5144)	(n = 367)	(n = 4777)
Male† - no. (%)	3496 (67.9)	314 (85.6)	3181 (66.6)
Mean age* - years (SD)	41.9 (18.3)	39.7 (15.9)	42.1 (18.5)
Mean age males - years (SD)	42.9 (18.0)	40.2 (15.7)	43.2 (18.2)
Mean age females - years (SD)	39.6 (18.8)	36.8 (17.3)	39.7 (18.9)
Travel history no. (%)	1392 (27)	179 (48.8)	1213 (25.4)
Europe	268	36	232
Asia	531	108	423
Africa	198	5	193
South America	153	11	142
Central and North America	102	16	86
Middle East	58	2	56
Australia	12	0	12
Unknown	70	1	69
DPO known - no. (%)	2733 (53.1)	338 (92.1)	2395 (50.1)
Single serology sample - no. (%)	4215 (81.9)	48 (13.1)	4167 (87.2)
Multiple serology samples - no. (%)	929 (18.1)	319 (86.9)	610 (12.8)
DPO 1^st^ serology sample# – median (IQR)	10 (5–22)	7 (5–11)	10 (5–24)
DPO follow up serology sample## – median (IQR)	24 (16–39)	21 (14–30)	28 (17–44)
Culture - no. (%)	1455 (28.3)	223 (60.8)	1232 (25.8)

† gender was registered for 5139 participants: 367 leptospirosis patients; 4772 non leptospirosis patients.

* Age was registered for 5143 participants: 367 leptospirosis patients; 4776 non leptospirosis patients.

# DPO of first sample was calculated from 2703 participants of whom first day of onset was known as well as date of sample collection: 330 leptospirosis patients; 2373 non leptospirosis patients.

## DPO of follow up sample was calculated from 630 participants of whom first day of onset was known as well as date of sample collection: 276 leptospirosis patients; 354 not leptospirosis patients.

**Table 2:** Diagnostic test and serogroup of Leptospirosis positive patients (n = 367).

Fullfillment of Case definition:	Multiple positive features, n = 282	Single positive feature, n = 85
Culture positive	31	6
MAT≥1∶160	253	20
IgM≥1∶160	234	45
Seroconversion MAT	140	4
Seroconversion IgM ELISA	108	10

Probable infecting serogroup*	Autochthonous cases, n = 188	Imported cases, n = 179
Grippothyphosa	27 (14.4%)	15 (8.4%)
Icterohaemorrhagiae	89 (47.3%)	33 (18.4%)
Other serogroups:		
Australis	1	8
Autumnalis	-	10
Ballum	2	1
Bataviae	-	6
Canicola	-	2
Celledoni	-	5
Cynopteri	-	2
Hebdomadis/Sejroe/Mini complex	-	15
Javanica	2	2
Pomona	14	1
Pyrogenes	-	4
Shermani	-	1
not classifiable	53 (28.2%)	74 (41.3%)

* Probable infecting serogroup is based on titers in MAT and typing results of positive cultures (Autumnalis n = 3, Bataviae n = 2, Canicola n = 2, Grippotyphosa n = 5, Hebdomadis n = 1, Icterohaemorrhagiae n = 19, Javanica n = 2, Pyrogenes n = 2, Shermani n = 1). Probable infecting serogroup could not be determined if patient was a case based only on IgM-ELISA or had several similar reacting serogroups in MAT.

RDTs were performed on 1^st^ and follow-up specimens from 861/929 participants (16.7% of all participants); 80.7% of the leptospirosis cases, and 11.8% of the non-leptospirosis participants. The total median number of days between 1^st^ and follow-up sample was 16 days (IQR 11 to 28). For the confirmed leptospirosis participants this was 14 days (IQR 8 to 22), versus 20 days (IQR 3 to 200) for the non-leptospirosis participants (P<0.05, Kruskal-Wallis test).

### Overall accuracy

The overall sensitivity and specificity, calculated on all samples from early acute till the late convalescent phase showed a sensitivity of 75% (95% CI 69% to 79%) for LeptoTek Dri Dot, 78% (95% CI 69% to 85%) for LeptoTek Lateral Flow and 78% (95% CI 71% to 83%) for Leptocheck-WB. The specificity was 96% (95% CI 95% to 97%) for LeptoTek Dri Dot, 95% (95% CI 94% to 96%) for LeptoTek Lateral Flow and 98% (95% CI 97% to 98%) for Leptocheck-WB (Table 3). There were no marked differences between the three tests; the sensitivities and specificities were similar with overlapping confidence intervals.

**Table 3:** Overall case sensitivity and specificity of rapid diagnostic tests.

Assay		Sensitivity	%	CI	Specificity	%	CI
LeptoTek Dri Dot	1st sample	131/256	51	45–57	2795/2903	96	96–97
	paired samples	137/167	82	76–87	261/286	91	87–94
	Any sample	194/259	75	69–79	2795/2909	96	95–97
LeptoTek Lateral Flow	1st sample	74/108	69	59–77	1235/1292	96	94–97
	paired samples	56/65	86	76–93	116/138	84	77–89
	Any sample	85/109	78	69–85	1229/1295	95	94–96
Leptocheck-WB	1st sample	100/183	55	47–62	2495/2551	98	97–98
	paired samples	103/129	80	72–86	162/174	93	88–96
	Any sample	153/197	78	71–83	2497/2560	98	97–98

### Accuracy of first sample and follow-up sample

When considering only the first sample that was sent in for each patient, the sensitivity of each test dropped dramatically from 75% to 51% and from 78% to 55% for the LeptoTek Dri Dot and the Leptocheck-WB, respectively. The sensitivity of the LeptoTek Lateral Flow decreased from 78% to 69%, although not a statistically significant change. The specificity of all tests remained more or less the same. Test results from paired samples (either one of the samples positive) increased the sensitivity significantly from 51% to 82% for the LeptoTek Dri Dot and from 55% to 80% for the Leptocheck-WB. The increase from 69% to 86% for the LeptoTek Lateral Flow was not statistically significant. The corresponding decrease in specificity was significant, i.e. from 96% to 91% for the LeptoTek Dri Dot, from 96% to 84% for the LeptoTek Lateral Flow and from 98 to 93% for the Leptocheck-WB (Table 3).

### Time trends

For 2733 participants (53.1% of study participants) the first day of onset of symptoms was known. All three tests show a lower sensitivity during the early acute phase of the disease (till DPO 4), which increased during DPO 5–10 and DPO 11–20, while the specificity of all tests remained relatively stable (Table 4). LeptoTek Lateral Flow was performing the best at DPO 0–4 (sensitivity of 62%, 95% CI 41% to 79% and specificity of 98%, 95 CI 93% to 99%).

**Table 4:** Sensitivity and specificity of rapid diagnostic tests at different days post onset (DPO).

Assay	DPO	Sensitivity	%	CI	Specificity	%	CI
LeptoTek Dri Dot	0–4		27	17–40		97	94–98
	5–10		55	47–63		96	94–98
	11–20		83	74–89		96	93–98
	>20		74	66–80		96	95–98
LeptoTek Lateral Flow	0–4		62	41–79		98	93–99
	5–10		75	62–84		94	89–96
	11–20		81	69–90		93	88–96
	>20		85	75–92		95	91–97
Leptocheck-WB	0–4		42	28–58		97	95–99
	5–10		65	55–74		96	94–97
	11–20		72	62–81		98	95–99
	>20		70	61–78		97	95–98

### Sensitivity analyses

The proportion of the indeterminate results for the 1^st^ sample for LeptoTek Dri Dot were 10/256 (4%) in the participants fulfilling the case definition and 85/2903 (3%) in the participants not fulfilling the case definition. For the LeptoTek Lateral Flow, these proportions were 4/108 (4%), respectively 173/1292 (13%), and for the Leptocheck- WB 17/183 (9%), respectively 239/2551 (9%).

**Figure 2:**
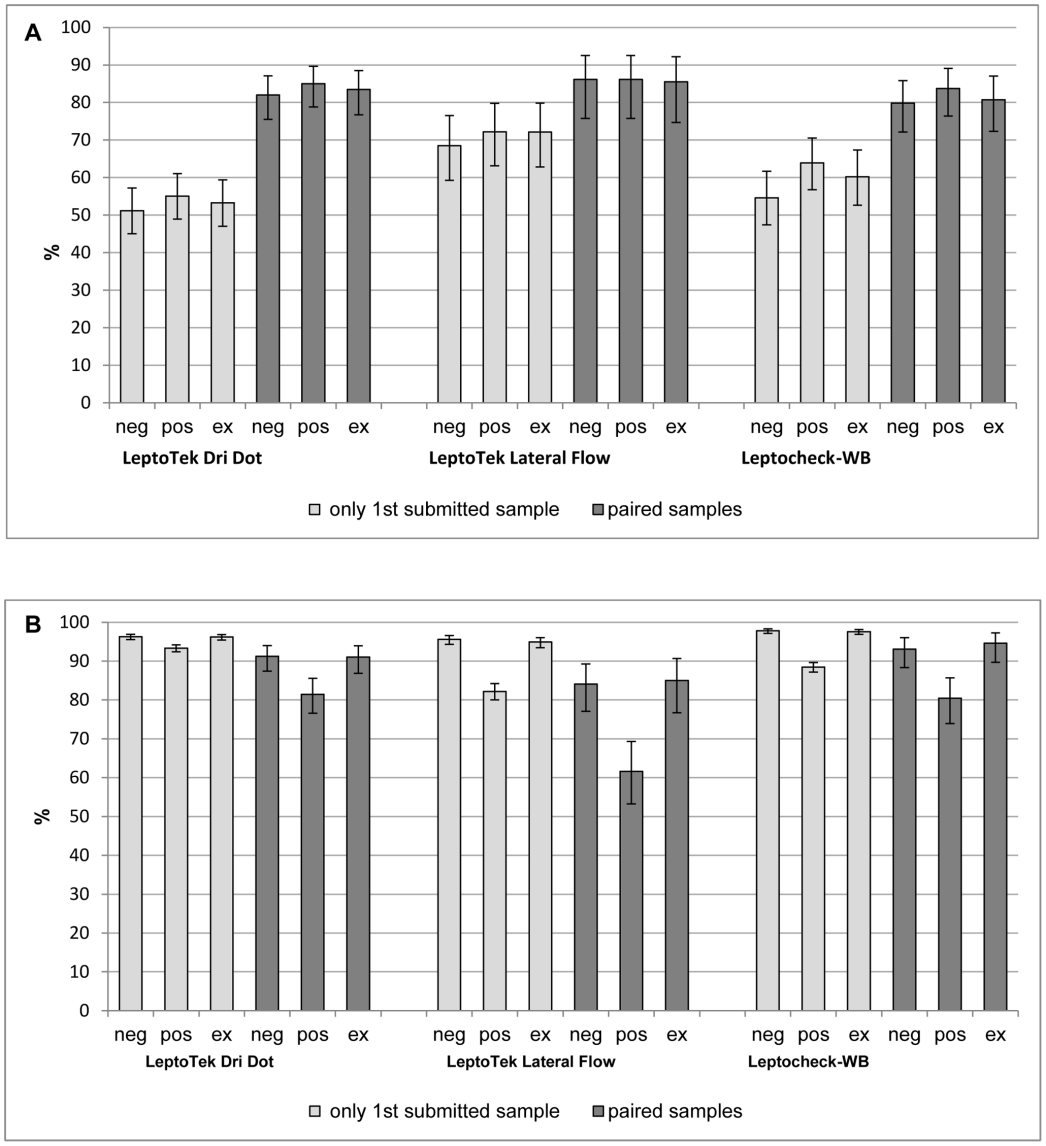
Sensitivity and specificity of the three RDTs of the 1^st^ submitted sample and paired samples. Intermediate results are considered either negative (neg) or positive (pos), or are excluded (ex). Panel A: sensitivity. Panel B: specificity.

Allocation of indeterminate results to positive scores did not substantially change sensitivity, but it did have an impact on specificity (Figure 2). For the LeptoTek Dri Dot, the specificity decreased from 96% to 93% for the 1^st^ submitted sample and from 91% to 81% for the paired samples. The LeptoTek Lateral Flow showed a decrease of the specificity from 96% to 82% for the 1^st^ sample and 84% to 62% for the paired samples, while the Leptocheck-WB showed a decrease from 98% to 88% and from 93% to 80% respectively.

About 28% of the participants with an initial indeterminate result for the LeptoTek Dri Dot and Leptocheck-WB were later confirmed with leptospirosis in follow-up testing, and had a positive RDT compared to only 10% of participants with an initial negative result. For the LeptoTek Lateral Flow, the numbers are somewhat different with 9% positive results after the first sample was indeterminate, and 4% positive results after the first sample was negative, but the same trend is present (Table S4).

Exclusion of indeterminate results showed an increasing sensitivity and decreasing specificity for all RDTs and for all time points, though not statistically significant. When stratifying the samples according to the defined time-periods of the disease, the same trend was observed (Table S3).

The sensitivity of RDTs appeared to depend on the infecting serogroup (Table S3). In general infecting serogroup Icterohaemorrhagiae yielded a higher sensitivity for all three RDTs compared to the other categories of serogroups. Differences were significant in the following cases: The LeptoTek Dri Dot showed a higher sensitivity for the paired samples in the Icterohaemorrhagiae infections (98%) compared to the other infections (81%) and non-classifiable serogroup infections (60%). The 1^st^ submitted samples of the latter category also had a lower sensitivity (38%) compared to the Icterohaemorrhagiae group infections (67%).

The LeptoTek Lateral Flow showed a higher sensitivity in both the 1^st^ submitted samples and the paired samples for the Icterohaemorrhagiae infections (85% respectively 100%) compared to for ‘non-classifiable serogroups’ (51% respectively 63%).

Leptocheck-WB showed a higher sensitivity in the 1^st^ samples (68%) as well as the paired samples (95%) for the Icterohaemorrhagiae infections compared to the category ‘non-classifiable serogroups’ (1^st^ submitted sample 38%, paired samples 65%).

### Temporal consistency

**Figure 3:**
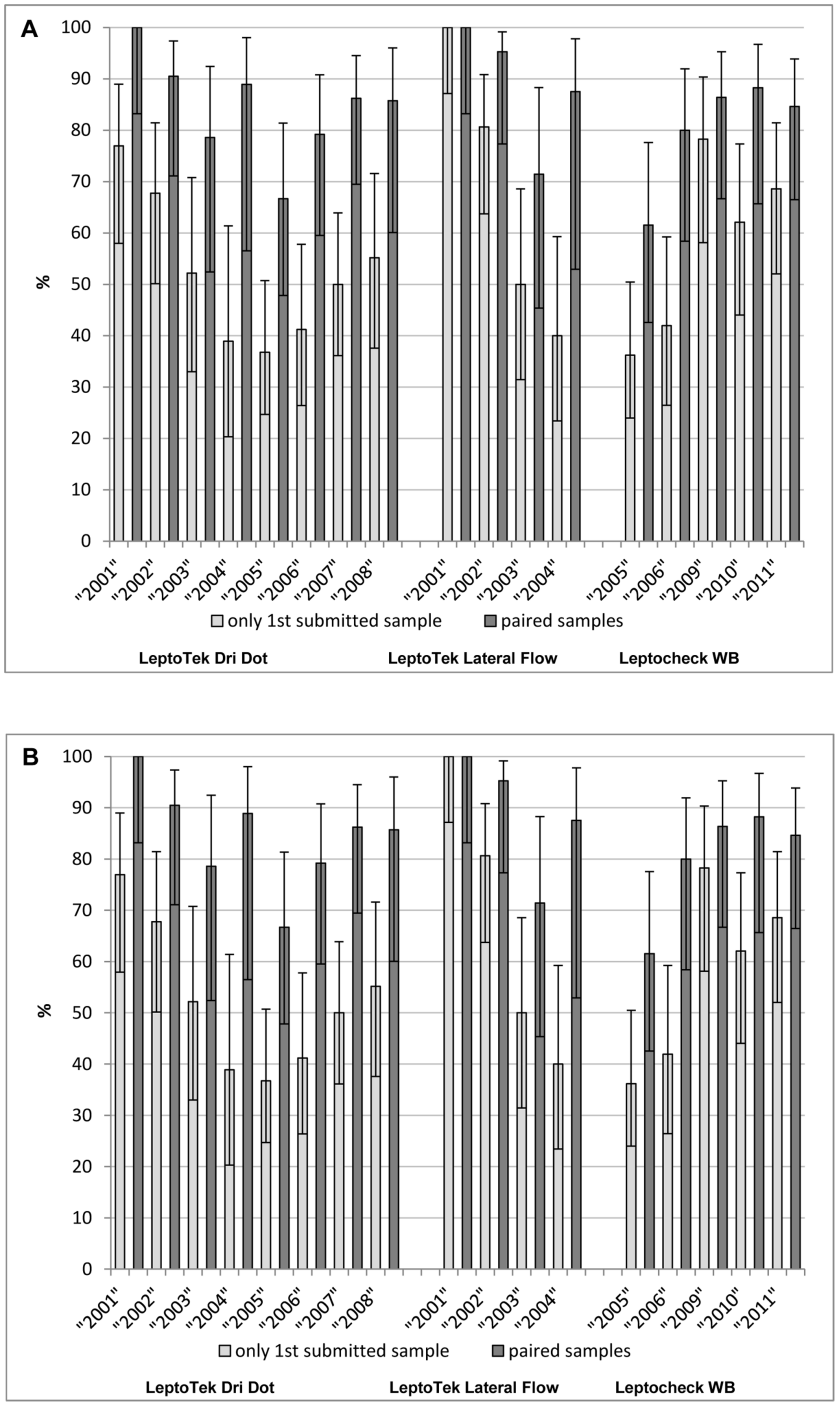
Sensitivity and specificity of the three RDTs of the 1^st^ submitted sample and paired samples. Results are presented for each year. Panel A: sensitivity. Panel B: specificity.

To investigate the consistency of the diagnostic accuracy of these RDTs over the time period 2001 to 2011, the diagnostic accuracy based on the 1^st^ submitted sample and paired samples for each year for each test was compared (Figure 3). Significant variation was observed in the following cases: for the 1^st^ sample submitted, the sensitivity of the LeptoTek Dri Dot decreased from 77% in 2001 to 37% in 2005 combined with increasing specificity from 93% to 98%. During the same years the paired samples showed a decrease in sensitivity from 100% to 67%. Also the LeptoTek Lateral Flow showed on the 1^st^ submitted sample a decreasing sensitivity from 100% in 2001 to 50% in 2003, whereas the specificity increased from 87% to 99%. For the paired samples, the specificity increased from 60% to 100%. On the contrary, based on the 1^st^ submitted sample the Leptocheck-WB showed an increase in sensitivity, from 36% in 2005 to 78% in 2009, combined with a decreasing specificity from 100% to 97%.

## Discussion

This paper presents data of a prospective evaluation of three RDTs for leptospirosis, the LeptoTek Dri Dot, the LeptoTek Lateral Flow and the Leptocheck-WB, on a well-defined Dutch population. The overall sensitivity and specificity did not vary much between the tests, with sensitivity ranging from 75% to 78% and specificity ranging from 95 to 98%.

However, when based on first submitted sample only, the sensitivity of all tests depreciated substantially, with corresponding specificities remaining high. The sensitivity of the LeptoTek Dri Dot and the Leptocheck-WB was markedly lower, i.e. 51% and 55%, respectively while the sensitivity of the LeptoTek Lateral Flow test dropped less to a still appreciable 69%. This low sensitivity of first sample can be explained by the fact that these samples usually are collected at an early stage of disease when antibodies are not present yet at detectable levels [15]. Consistently, the sensitivity of the three tests increased to more than 80% when results of a follow-up sample were included, supporting a significant increase of sero-diagnostic sensitivity when using paired samples as previously reported [10], [15]. However, as the sensitivity increased with paired samples, the concomitant specificity reduced, with the largest reduction found for the LeptoTek Lateral Flow, i.e. from 96% to 84%.

Clinically this indicates that with around 500 suspected cases annually received at the NRL, comprising approximately 30 confirmed leptospirosis cases, RDTs used on the first sample alone, would lead to between half (LeptoTek Dri Dot, sensitivity of 51%) and a third (LeptoTek Lateral Flow) of the cases being missed. Yet, if paired samples are considered, then only 4 to 6 confirmed leptospirosis patients would be missed. This strongly advocates for clinicians to provide follow-up samples [15]. These paired samples, however, increase the number of false positives (from 17 to 33–75), which might contribute to an unneeded continuation of treatment with antibiotics.

It should be pointed out that in most situations, where leptospirosis is highly endemic, availability of only one acute phase sample is common practice and, hence, the diagnostic accuracy of tests on early acute samples is most relevant. In general, these RDTs showed disappointingly low sensitivities at the early stage of the disease, although associated with acceptable specificities of around 97%. From the subgroup analysis involving samples with known DPO, the LeptoTek Lateral Flow Test presents a favorable exception. Its sensitivity in the early acute phase was 62%, which is significantly higher than the sensitivity of the Leptocheck-WB (27%) and the LeptoTek Dri Dot (42%). From the literature, 62% is also higher than usually reported on the sensitivity of the MAT and ELISA in the earliest stage of the disease [10], [15], [19]. Apparently, this test is more capable to effectively detect ‘early’ antibodies and, hence, presents a respectable adjunct laboratory tool enabling a timely start of adequate care handling of patients.

This higher sensitivity in the early acute phase cannot be explained by the choice of antigen, which is most likely similar, since both lateral flow assays (LFAs) have been constructed using crude antigen probably derived from *Leptospira biflexa*, serovar Patoc, strain Patoc I [20]. However, differences in the diagnostic accuracy between the two LFAs might be caused by differences in the production, such as applying different amounts of antigen to the LFAs or providing different quantities of conjugate. Of note, visual inspection albeit subjective, judged the staining of the band in the LeptoTek Lateral Flow test stronger than that of the Leptocheck-WB (unpublished observation) facilitating an easier positivity score. Differences found in the diagnostic accuracy of the LeptoTek Dri Dot can easily be explained by the use of a different antigen that consists of crude antigen derived from *L. borgpetersenii*, serovar Hardjo type Bovis, strain Lely 607 [21], although differences in production procedures might remain a valid explanation.

All RDTs showed a lower specificity when testing paired samples compared to the 1^st^ submitted sample only. A possible explanation is that cases from whom follow-up samples were received more frequently present with persistent complaints due to chronic disorders such as autoimmune diseases that are notorious for causing cross-reactions in serological assays. However, be aware that in general, the more tests one does, the more likely the tests will be positive (which can lead to an increase in false positives). In this study, we have seen that for all three RDTS as sensitivity rises, specificity convergently decreases.

An unexpected high percentage of indeterminate results were found, considering the fact that the reading of the RDTs was done on a daily basis by a small group of well-experienced staff. This indicates that these tests are not always easily read. Although we found a high proportion of tests results to be indeterminate, especially for the LeptoTek Lateral Flow, this does not imply that such indeterminate results are of no value. In the sensitivity analyses we saw that scoring all indeterminate results as positive (instead of negative or excluded from analyses) resulted in an increase in the sensitivity of a test, as expected, but this corroborated with an unwanted reduction of test specificity, depending on the proportion of indeterminate results. Therefore, there may be practical consequences of a decision to score an indeterminate result as positive or negative. In case of a first sample giving an indeterminate result, a clinician might choose to regard this result as a negative result and not send in a follow-up sample. However, we have seen in those patients with follow-up samples, that in about 28% of the cases where the first sample gave an indeterminate LeptoTek Dri Dot or Leptocheck-WB result, the follow up sample gave a positive result and likely is a true positive, compared to only 10% of the first samples with a negative outcome. Hence, this implies that the indeterminate results incline more towards a final true case than negative results. Apart from the fact that a follow-up sample always should be issued after initial negative outcome, it is particularly advised to submit a follow-up specimen in case of an indeterminate result.

From the additional diagnostic accuracy of the RDTs by infecting serogroup, the sensitivity of all three RDTs was higher for infection with the Icterohaemorrhagiae group compared to infections with other serogroups. There is no conclusive explanation why infections with the other categories are associated with lower sensitivity. It may be that patients infected with Icterohaemorrhagiae present with more severe disease and may elicit strong humoral responses [22]. The finding that the test sensitivity depends on the causative leptospires associated with that fact that there is a wide diversity of geographic distribution of most *Leptospira* serovars suggests that the diagnostic accuracy of the various tests most likely will vary in different geographical locations. This explains, at least in part, the discrepant results of RDTs in previous studies performed in various regions [9], [10] and reiterates that it is imperative to do a local evaluation and validation of tests prior to implementation.

The results revealed that the diagnostic accuracy of the RDTs varies through the years of our study. The sensitivity of the LeptoTek Lateral Flow and LeptoTek Dri Dot tended to decrease during the years while the Leptocheck-WB increased in sensitivity. Although it is unclear why this variability is present, it implies that one cannot rely on a constant performance of commercial RDTs, hence emphasizing the importance of continuous and thorough quality control of the RDTs by the manufacturer. Moreover, for the user this necessitates the evaluation of new purchases, preferably by using a standardized set of sera comprising a range of low to high ‘reactors’.

The validity of our data is positively affected by the prospective nature of the evaluation as well as the use of fresh specimens. Furthermore, all participants suspected for leptospirosis were included in the study, allowing those who did not meet the case definition to serve as controls, hence evading the use of a less realistic, separate sample ‘healthy controls’ [23]. The case definition in this study was based both on culture and serology (MAT and IgM-ELISA). The MAT has a disappointing low sensitivity in the early phase of infection [15], [24] and consequently, reliance on only the MAT as reference standard would result in a proportion of incorrect false positive scores for the RDTs with an erroneous lower specificity. The RDTs were performed by well-trained staff who were used to (optically) reading the tests results and performed prior to serologic tests and culture.

The study also presents with limitations. Only a subgroup of participants had their first day of illness documented and documentation on both treatment and hospitalization was not available. This information could have affected the test results, since it is known that the use of antibiotics reduces the immune response. Follow-up samples were not received from all participants. Therefore what is considered a false positive RDT result could actually have turned out to be leptospirosis cases, if a confirmatory sample had been received. With a high level of indeterminate tests, the study misses information on repeatability or reproducibility.

### Conclusion and recommendations

The LeptoTeK Lateral Flow presents in all scenarios with the best sensitivity and equally good specificity of all three RDT tests. All three tests, LeptoTek Dri Dot, LeptoTek Lateral Flow and Leptocheck-WB present useful antibody tests contributing to the diagnosis of leptospirosis. For sure, confirmation of clinical suspicion will contribute to increased local awareness of leptospirosis. Confirmation might also be beneficial for the clinical management of the patient. On the other hand, it should be noted that, especially in the early phase, a negative RDT and a high clinical suspicion still warrants antibiotic treatment since (untreated) leptospirosis is a potential fatal disease. Unfortunately, currently LeptoTek Dri Dot and LeptoTek Lateral Flow are not available due to manufacturer issues, presently leaving few options. The overall sensitivity of the tested RDTs did not exceed 80%, while their performance might depend on batch-to-batch and year-to-year variations as well as on varying ecological niches containing different circulating serovars. This latter drawback might be extended with a reduced diagnostic accuracy due to past leptospiral infections or infections with other causative agents in high endemic areas, causing cross-reactions in these tests [9]. For these reasons, one should be cautious to only rely on an RDT result. Confirmation by reference tests is strongly recommended, and further conclusive studies are needed in endemic regions. From this study we have seen that rapid testing is not synonymous with easy testing. Reading of tests by eye is subjective and depends on the experience of the reader. At least it is of great importance that a test result, in case of doubt, is reported as such, indicating the need for a follow-up sample, especially evading the inclination of the reader to score a doubtful signal as a positive score.

## Supporting Information

Table S1 **STARD checklist for reporting studies of diagnostic accuracy.**
(DOC)

Table S2 **Availability of the three RDTs throughout the years.**
(TIF)

Table S3 **Results of the three RDTs.** The results are stratified for: 1^st^ sample and follow up (FU) sample, DPO (0–4, 5–10, 11–20, >20), probable infecting serogroup (Icterohaemorrhagiae, Grippotyphosa, other, non-classifiable), years.
(TIF)

Table S4 **Predictive value of indeterminate and negative test results.**
(TIF)
