# Supplementary figures and images for: Prospective Evaluation of Three Rapid Diagnostic Tests for Diagnosis of Human Leptospirosis
Source: PLoS Negl Trop Dis. 2013 Jul 11;7(7):e2290. doi: 10.1371/journal.pntd.0002290 (PMC3708816; doi:10.1371/journal.pntd.0002290)

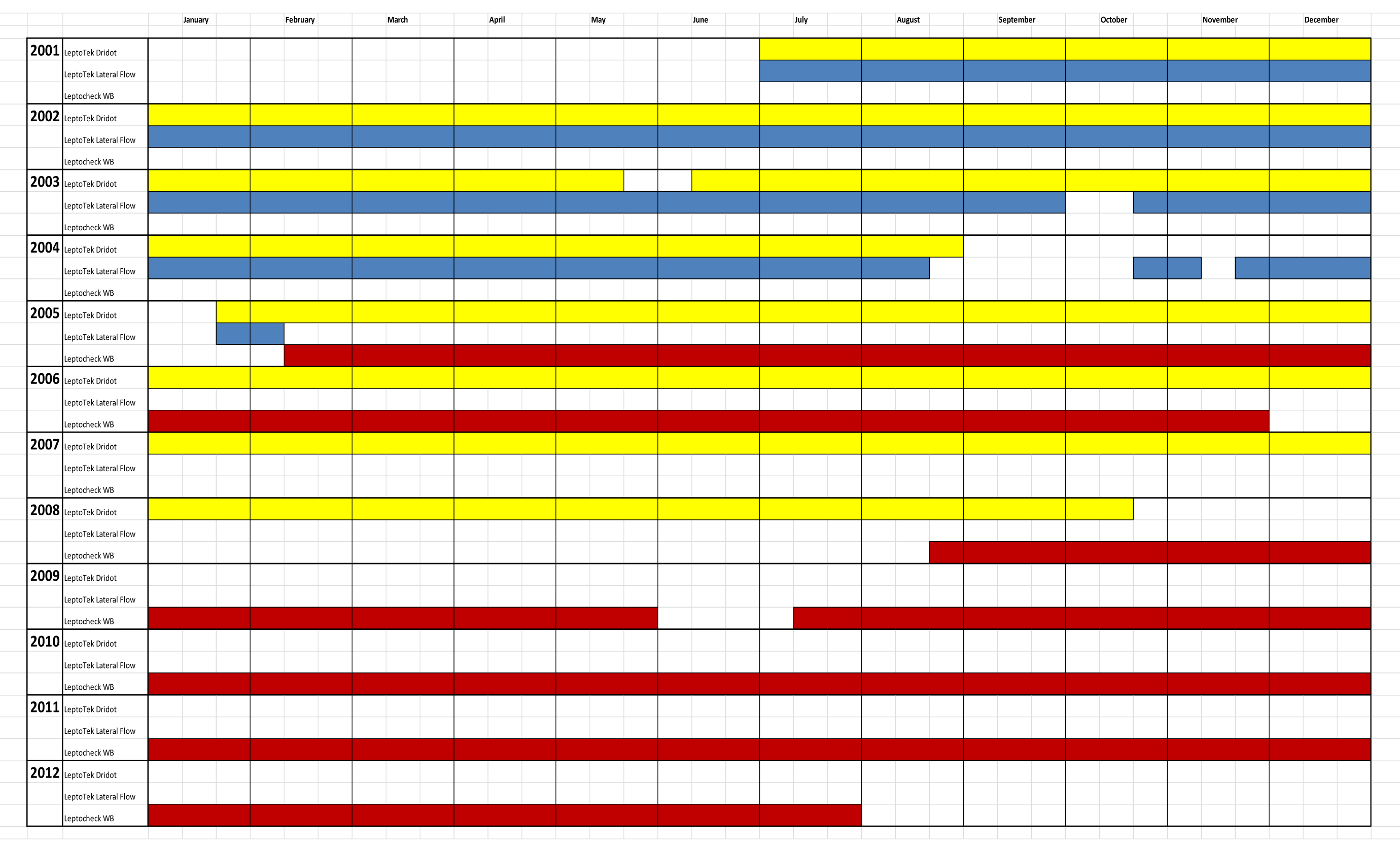

Supplement: Table S2 — Availability of the three RDTs throughout the years. (TIF) [file pntd.0002290.s002.tif]

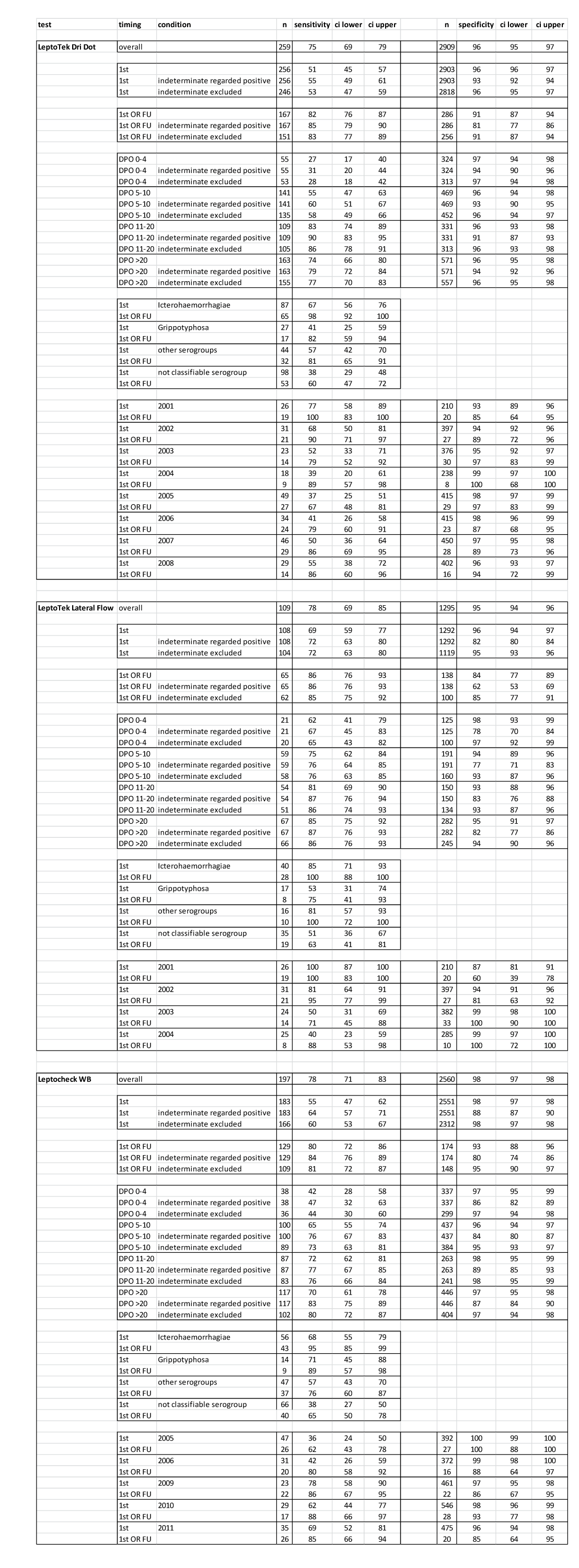

Supplement: Table S3 — Results of the three RDTs. The results are stratified for: 1st sample and follow up (FU) sample, DPO (0–4, 5–10, 11–20, >20), probable infecting serogroup (Icterohaemorrhagiae, Grippotyphosa, other, non-classifiable), years. (TIF) [file pntd.0002290.s003.tif]

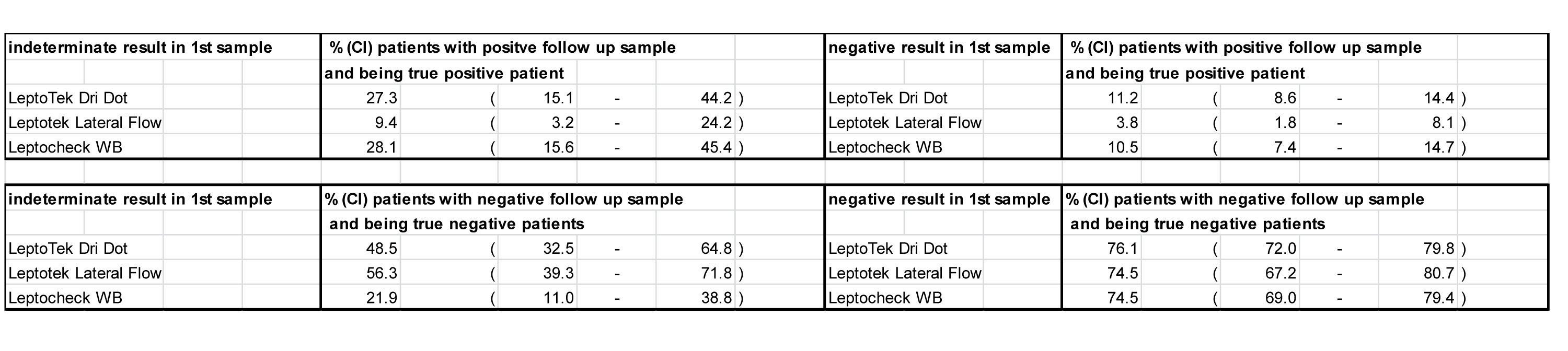

Supplement: Table S4 — Predictive value of indeterminate and negative test results. (TIF) [file pntd.0002290.s004.tif]
